# Sex differences in the coexpression of prokineticin receptor 2 and gonadal steroids receptors in mice

**DOI:** 10.3389/fnana.2022.1057727

**Published:** 2023-01-06

**Authors:** Brenda Cisneros-Larios, Carol Fuzeti Elias

**Affiliations:** ^1^Department of Molecular and Integrative Physiology, University of Michigan, Ann Arbor, MI, United States; ^2^Elizabeth W. Caswell Diabetes Institute, University of Michigan, Ann Arbor, MI, United States; ^3^Department of Gynecology and Obstetrics, University of Michigan, Ann Arbor, MI, United States; ^4^Neuroscience Graduate Program, University of Michigan, Ann Arbor, MI, United States

**Keywords:** sex differences, posterior amygdala, estrogen, androgen, prokineticin

## Abstract

Loss-of-function mutations in prokineticin 2 (*PROK2*) and the cognate receptor prokineticin receptor 2 (*PROKR2*) genes have been implicated in reproductive deficits characteristic of Kallmann Syndrome (KS). Knock out of *Prokr2* gene produces the KS-like phenotype in mice resulting in impaired migration of gonadotropin releasing hormone (GnRH) neurons, olfactory bulb dysgenesis, and infertility. Beyond a developmental role, pharmacological and genetic studies have implicated PROKR2 in the control of the estrous cycle in mice. However, PROKR2 is expressed in several reproductive control sites but the brain nuclei associated with reproductive control in adult mice have not been defined. We set out to determine if ProkR2 neurons in both male and female mouse brains directly sense changes in the gonadal steroids milieu. We focused on estrogen receptor α (ERα) and androgen receptor (AR) due to their well-described function in reproductive control *via* actions in the brain. We found that the ProkR2-Cre neurons in the posterior nucleus of the amygdala have the highest colocalization with ERα and AR in a sex-specific manner. Few colocalization was found in the lateral septum and in the bed nucleus of the stria terminalis, and virtually no colocalization was observed in the medial amygdala. Our findings indicate that the posterior nucleus of the amygdala is the main site where PROKR2 neurons may regulate aspects of the reproductive function and social behavior in adult mice.

## Introduction

A spectrum of reproductive deficits and anosmia, or loss of smell, are characteristics of patients diagnosed with Kallmann Syndrome (KS). Complex genetic studies in humans as well as data from mouse models have identified several genes associated with this clinical condition (Hardelin and Dodé, 2008). Among them, a series of distinct mutations in the prokineticin receptor 2 (*PROKR2*) gene have been identified in KS patients (Abreu et al., 2008; Martin et al., 2011; Avbelj Stefanija et al., 2012). *Prokr2* knockout mice reproduce the phenotype of patients with KS, as they show severe defects in olfactory bulb development, a significant decrease in the numbers of gonadotropin releasing hormone (GnRH) neurons in the medial septum-preoptic area, and a decrease in GnRH fibers in the median eminence (Ng et al., 2005; Matsumoto et al., 2006).

While the loss of function mutation in both *Prokr2* alleles results in impaired GnRH neuronal migration, *Prokr2*-null heterozygous mice show a regular number of GnRH neurons within the hypothalamus suggesting normal migration. Interestingly, these mice exhibit a disrupted estrous cycle indicating that, despite normal GnRH migration, the reproductive function is compromised (Xiao et al., 2014). However, the brain sites associated with the role of PROKR2 neurons in adult reproduction are not known.

Initial studies in male mice have shown that *Prokr2* mRNA is expressed in several reproductive control sites, including the lateral septum, preoptic area, and arcuate nucleus, and strong expression in the medial nucleus of the amygdala (Cheng et al., 2006). Neurons of the lateral septum, preoptic area, and medial amygdala integrate olfactory stimuli and mediate the appropriate reproductive endocrine and behavioral responses (Baum et al., 1982; DiBenedictis et al., 2012; Baum and Cherry, 2015).

With the aim of increasing the experimental tools to assess the role of PROKR2 in adult reproduction, we developed a mouse model expressing Cre recombinase driven by the *Prokr2* promoter (Mohsen et al., 2017). Using the ProkR2-Cre reporter mouse, we reproduced the previous findings in males and expanded the data in females (Mohsen et al., 2017). These findings outlined the distribution of neuronal *Prokr2* and putative roles in reproductive physiology and sexual behavior guided by prokineticin actions in the brain. Although the expression profile of *Prokr2* in male and female brains has been characterized, it is not clear if subpopulations of neurons sense changing levels of circulating gonadal steroids required for the estrous cycle and social behavior.

Therefore, to begin assessing potential brain sites where PROKR2 cells influence the reproductive axis, we performed a systematic evaluation of the coexpression of estrogen receptor-α (ERα) and androgen receptor (AR) with ProkR2-Cre neurons in male and female brains using the ProkR2-Cre mouse model (Mohsen et al., 2017). We focused on ERα and AR due to their well-described role in adult reproduction in both sexes (Lisk and Bezier, 1980; Rasia-Filho et al., 1991; Wood et al., 1992; Lubahn et al., 1993; Wood and Newman, 1993, 1995a; Eddy et al., 1996; Wood, 1996; Wood and Coolen, 1997; Yeh et al., 2002; Sato et al., 2004; Juntti et al., 2010; Wang et al., 2018).

## Materials and Methods

### Animals

Adult (2–5 months old) male and female ProkR2-Cre mice were used for all experiments (JAX^®^ stock #007914; Mohsen et al., 2017). The ProkR2-Cre mice were crossed with Cre-dependent, eGFP-L10a mice kindly provided by Dr. David Olson at the University of Michigan (Krashes et al., 2014) and commercially available (JAX^®^ stock #024750). All mice were heterozygous for the ProkR2-Cre allele with one or two copies of the eGFP-L10a allele. Mice were maintained in a light- (12L:12D) and temperature- (21°C–23°C) controlled environment at the University of Michigan Animal Facility (ULAM). Animals had free access to water and a phytoestrogen-reduced Envigo diet 2016 (16% protein/4% fat). A phytoestrogen-reduced diet was used to minimize the effects of exogenous estrogen in the expression of gonadal steroids receptors (Boettger-Tong et al., 1998; Brown and Setchell, 2001; Thigpen et al., 2004; Dinsdale and Ward, 2010). All procedures and experiments were done in accordance with guidelines established by the National Institutes of Health Guide for the Care and Use of Laboratory Animals and approved by the Institutional Animal Care and Use Committee (IACUC) at the University of Michigan (protocol # PRO00010420).

All mice were genotyped as outlined in Mohsen et al. (2017).

### Perfusion and brain histology

Mice were anesthetized with isoflurane and transcardially perfused with 0.1 M PBS followed by 10% neutral buffered formalin. All perfusions were done between 12:00 noon and 2:00 PM (corresponding to ZT6 and ZT8). Following perfusion, brains were collected and postfixed in 20% sucrose-10% formalin for 2 h. Brains were cryoprotected in 20% sucrose-PBS and sectioned in a freezing microtome (Leica). Four series of 25-μm thick coronal sections were collected and stored at −20°C in cryoprotectant.

### Dual label immunoperoxidase

Sections were washed with 0.1 M PBS to remove cryoprotectant and incubated in 0.3% hydrogen peroxide (H_2_O_2_) in Triton-PBS (0.25%) for 30 min. Sections were then washed in 0.1 M PBS and were incubated in the primary antibody. The following antibodies and concentrations were used: rabbit anti-estrogen receptor-α (ERα) primary antibody (1:20,000, Millipore Cat# 06-935, RRID:AB_310305) and rabbit anti-androgen receptor (AR) antibody (1:400, Abcam Cat# ab133273, RRID:AB_11156085). All primary incubation were prepared with 3% donkey serum in Triton X100—0.1 M PBS (0.25%) and tissues were incubated overnight at room temperature. The following day, sections were washed in 0.1 M PBS and incubated in secondary biotin-conjugated donkey anti-rabbit antibody for 1 h (1:1,000, Jackson ImmunoResearch Laboratories), washed again in 0.1 M PBS and incubated in Avidin-Biotin Complex (ABC) in 0.1 M PBS (1:500, Vector Labs) for 1 h. The peroxidase reaction was performed using 0.05% 3,3’-diaminobenzidine (DAB, Sigma) and 0.05% nickel ammonium sulfate (Nickel, Sigma) as chromogens and 0.01% hydrogen peroxidase. Following 0.1 M PBS washes and another 30 min 0.3% hydrogen peroxide (H_2_O_2_) incubation, sections were incubated in chicken anti-GFP primary antibody (1:20,000, AvesLabs, catalog #GFP-1010; RRID:AB_2307313) with 3% donkey serum in Triton-0.1 M PBS (0.25%). The following day, the same steps previously described were followed except that a secondary biotin-conjugated donkey anti-chicken antibody (1:1,000, Jackson ImmunoResearch Laboratories) was used and Nickel was omitted from the DAB solution. Sections were mounted on gelatin-coated slides, dehydrated, delipidated in xylenes, for 15 min and coverslipped with DPX (EMS, Hatfield, PA).

A series of brain sections were counterstained with thionin for anatomical references. Slides were dipped in 0.25% thionin for 1 min, dehydrated, delipidated in xylenes for 15 min, and coverslipped with DPX (EMS, Hatfield, PA).

### Imaging and data analysis

Brain regions were evaluated using the digital Allen Mouse Brain atlas and sites showing colocalization of AR or ERα with ProkR2-Cre eGFP-L10a positive cells were identified. Quantification of dual labeled neurons was performed using ImageJ cell counter plug-in and Graphpad was used for data analysis. Only cells showing clear nuclear staining were deemed as dual labeled. Subjective analysis comparing dual labeled neurons based on relative expression (e.g., no dual labeled neurons visualized = −; highest dual labeled neurons visualized = +++) was performed by two independent evaluators. To determine potential sex differences in the number of neurons in the PA, we quantified the most posterior level of the PA (where we see the highest colocalization of ProkR2-Cre eGFP and AR or ERα) using thionin staining. Only one section and one side of the amygdala were quantified/mice. All blue neurons and dark brown (blue + eGFP labeled) were quantified. Microphotographs were acquired using the upright microscope Zeiss Axio Imager M2 and a digital camera (Axiocam, Zeiss, Germany) using the Zen software. Images were organized in figures using Adobe Photoshop. Brightness, sharpness, and contrast were adjusted for image presentation.

### Statistics

Analysis was performed using Prism, version 9 (GraphPad Software Inc). Data are reported as the mean ± SEM. The normal distribution of data was determined using a Shapiro-Wilk test (significance alpha 0.05). An unpaired *t*-test with Welch’s correction was used to analyze data. *P* < 0.05 was considered statistically significant. Data are reported as the mean ± SEM.

## Results

We performed a systematic evaluation of the ProkR2-Cre L10eGFP neurons that co-express AR or ERα in male and female brains (Table 1, *n* = 5 males for AR and ERα, *n* = 3 females for AR and *n* = 6 females for ERα). Because levels of estradiol may alter *Esr1* expression in opposite ways in different nuclei (Liu and Shi, 2015), we used cycling females (undefined estrous cycle stage) to avoid the effect of excess (E2 treatment) or lack (ovariectomy) of estradiol in specific nuclei. We focused our analysis on the forebrain where virtually all reproductive control sites are located.

**Table 1:** Subjective analysis of the coexpression of ProkR2-Cre eGFP and androgen receptor (AR) or estrogen receptor α (ERα) immunoreactivity in male and female brains.

**Brain areas and nuclei**	**Atlas Image**	**Male**		**Female**	
		**AR**	**ERα**	**AR**	**ERα**
Lateral septal nucleus, rostral (LSr)	53	+	−	+	±
Medial preoptic nucleus (MPN)	55	+	−	±	±
Bed nucleus of stria terminalis, posterior (BSTp)	56	±	−	±	±
Suprachiasmatic nucleus (SCH)	59	−	−	±	−
Paraventricular nucleus of hypothalamus (PVH)	61	+	±	±	+
Arcuate nucleus (ARH)	69	−	−	±	±
Medial nucleus of the amygdala, posterodorsal (MEApd)	70	−	−	−	−
Ventromedial nucleus, ventrolateral (VMHvl)	70	−	−	+	+
Ventral premammillary nucleus (PMv)	76	−	−	±	−
Posterior nucleus of the amygdala (PA)	80	+++	+++	+	+++

Only Notations −, ±, +, and +++ represent absent, very low, low, and high colocalization. The Allen Mouse Brain atlas was used as a reference for brain nuclei and localization (atlas image).

### Distribution of AR and ERα in ProkR2-Cre eGFP neurons of male mice

**Figure 1:**
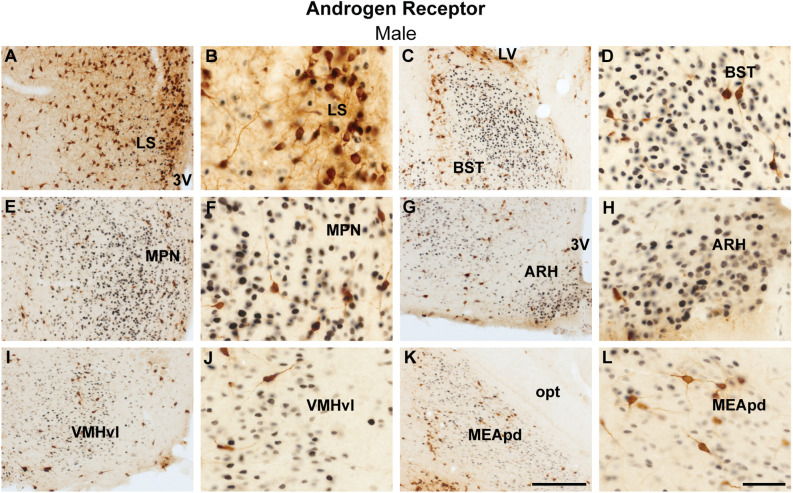
Coexpression of ProkR2-Cre eGFP and androgen receptor (AR) in the forebrain of the male mouse. **(A–L)** Bright field micrographs showing the expression of AR (black) and GFP (brown) immunoreactivity in the lateral septal nucleus, LS **(A,B)**, the bed nucleus of stria terminalis, BST **(C,D)**, the medial preoptic nucleus, MPN **(E,F)**, the arcuate nucleus, ARH **(G,H)**, the ventromedial hypothalamic nucleus, ventrolateral subdivision, VMHvl **(I,J)**, the medial nucleus of the nucleus, and posterodorsal subdivision, MEApd **(K,L)**. Abbreviations: LV, lateral ventricle; opt, optic tract; 3 V, third ventricle. Scale Bars: **(A,C,E,G,I,K)** = 200 μm; **(B,D,F,H,J,L)** = 50 μm.

The analysis of colocalization of AR or ERα with ProkR2-Cre eGFP was focused on forebrain nuclei previously described to express *Prokr2* mRNA and the reporter gene (Cheng et al., 2006; Mohsen et al., 2017). The lateral septum and the bed nucleus of the stria terminalis had minimal colocalization of ProkR2-Cre eGFP and AR (Figures 1A–D). Virtually no colocalization of Prokr2 GFP with AR was found in the suprachiasmatic nucleus (Table 1). We also found minimal colocalization in the medial preoptic nucleus (Figures 1E,F), paraventricular nucleus of the hypothalamus (Table 1), arcuate nucleus (Figures 1G,H), and ventral premammillary nucleus (Table 1). No colocation of ProkR2-Cre eGFP and AR was observed in the ventromedial hypothalamic nucleus (Figures 1I,J). In the amygdala, virtually no colocalization was observed in the posterodorsal subdivision of the medial nucleus (Figures 1K,L). However, high numbers of ProkR2-Cre eGFP immunoreactive cells of the posterior amygdala (PA, a.k.a., amygdalohypothalamic area or AHi) coexpress AR (Tables s 1 and 2).

**Table 2:** Quantification of cells coexpressing ProkR2-Cre eGFP and androgen receptor (AR) or estrogen receptor α (ERα) immunoreactivity in the posterior nucleus of the amygdala of male and female mouse brains.

**AR**	**Total GFP-ir**	**Total AR-ir**	**% Dual labeled /Total GFP-ir**	**% Dual labeled/Total AR-ir**
Male	323.4 ± 17.57	111 ± 32.88	24.7 ± 6.8%	70.6 ± 7.5%
Female	176 ± 23.29	33.33 ± 11.62	4.5 ± 1.8%	22.3 ± 4.3%
ERα	Total GFP-ir	Total ERα-ir	% Dual labeled/Total GFP-ir	% Dual labeled/Total ERα-ir
Male	333.2 ± 28.5	100.2 ± 10.39	21.8 ± 2.6%	71.8 ± 5.5%
Female	193.67 ± 12.93	89.67 ± 15.28	23.0 ± 3.5%	53.8 ± 7.4%

**Figure 2:**
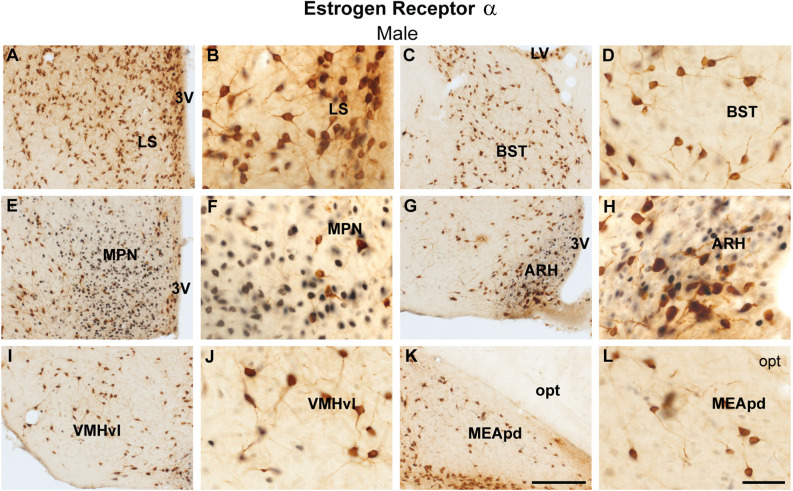
Coexpression of ProkR2-Cre eGFP and estrogen receptor α (ERα) in the forebrain of the male mouse. **(A–L)** Bright field micrographs showing the expression of ERα (black) and GFP (brown) immunoreactivity in the lateral septal nucleus, LS **(A,B)**, the bed nucleus of stria terminalis, BST **(C,D)**, the medial preoptic nucleus, MPN **(E,F)**, the arcuate nucleus, ARH **(G,H)**, the ventromedial hypothalamic nucleus, ventrolateral subdivision, VMHvl **(I,J)** and the medial nucleus of the amygdala, posterodorsal subdivision, MEApd **(K,L)**. Abbreviations: LV, lateral ventricle; opt, optic tract; 3 V, third ventricle. Scale Bars: **(A,C,E,G,I,K)** = 200 μm; **(B,D,F,H,J,L)** = 50 μm.

Similarly, virtually no colocalization between ProkR2-Cre eGFP and ERα was found in the lateral septum, bed nucleus of the stria terminalis, medial preoptic area, arcuate nucleus, ventromedial hypothalamic nucleus, and posterodorsal subdivision of the medial amygdala (Figures 2A–L). Moderate colocalization with ProkR2-Cre eGFP was also observed in the PA (Tables s 1 and 2).

### Distribution of AR and ERα in ProkR2-Cre eGFP neurons of female mice

**Figure 3:**
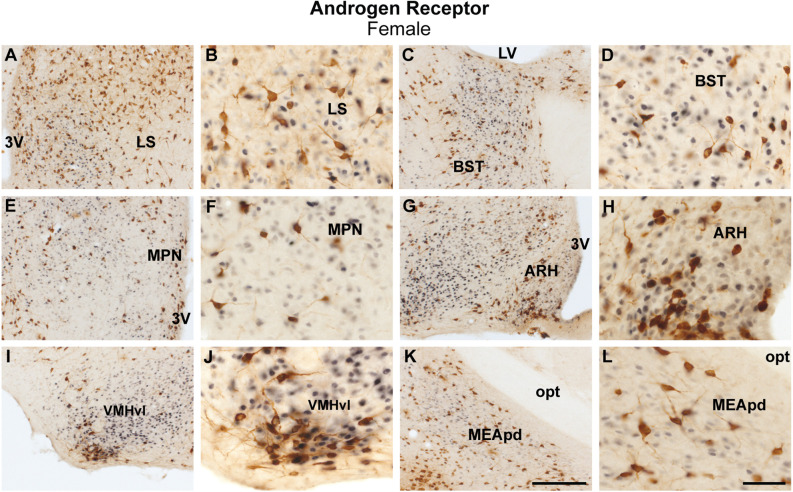
Coexpression of ProkR2-Cre eGFP and androgen receptor (AR) in the forebrain of the female mouse. **(A–L)** Bright field micrographs showing the expression of AR (black) and GFP (brown) immunoreactivity in the lateral septal nucleus, LS **(A,B)**, the bed nucleus of stria terminalis, BST **(C,D)**, the medial preoptic nucleus, MPN **(E,F)**, the arcuate nucleus, ARH **(G,H)**, the ventromedial hypothalamic nucleus, ventrolateral subdivision, VMHvl **(I,J)**, and the medial nucleus of the amygdala, the posterodorsal subdivision, MEApd **(K,L)**. Abbreviations: LV, lateral ventricle; opt, optic tract; 3 V, third ventricle. Scale Bars: **(A,C,E,G,I,K)** = 200 μm; **(B,D,F,H,J,L)** = 50 μm.

Female brains showed a comparable coexpression pattern to male brains. The lateral septum had minimal colocalization between ProkR2-Cre eGFP and AR, while sporadic colocalization was observed in the bed nucleus of stria terminalis (Figures 3A–D). No colocalization between ProkR2-Cre eGFP and AR was found in the suprachiasmatic nucleus, and minimal colocalization was observed in the medial preoptic nucleus, paraventricular hypothalamus, and arcuate nucleus (Figures 3E–H, Table 1). Very few ProkR2-Cre eGFP cells colocalize AR in the ventromedial hypothalamus (Figures 3I,J). We found virtually no colocalization of ProkR2-Cre eGFP and AR in the posterodorsal subdivision of the medial amygdala (Figures 3K,L). Small coexpression of ProkR2-Cre eGFP and AR was observed in female PA (Tables s 1 and 2).

Colocalization of ProkR2-Cre eGFP with ERα was briefly reported by our lab (Mohsen et al., 2017). In a more comprehensive analysis, in this study, we reproduced our initial findings and found no colocalization of ProkR2-Cre eGFP and ERα in the bed nucleus of the stria terminalis and arcuate nucleus (Table 1), and very low colocalization in the ventromedial nucleus of the hypothalamus (Table 1). In addition, we found very low colocalization of ProkR2-Cre eGFP and ERα in the paraventricular nucleus of the hypothalamus (Table 1). Higher colocalization was observed in the PA (Tables s 1 and 2).

### Colocalization of ProkR2-Cre eGFP with AR or with ERα in the posterior nucleus of the amygdala (PA) shows sex differences

The PA is the forebrain site with the highest colocalization of ProkR2-Cre eGFP and AR or ERα. Because in previous studies we found sex differences in ProkR2-Cre eGFP expression (Mohsen et al., 2017), we performed a careful quantification of colocalization to compare the findings between sexes (Table 2).

**Figure 4:**
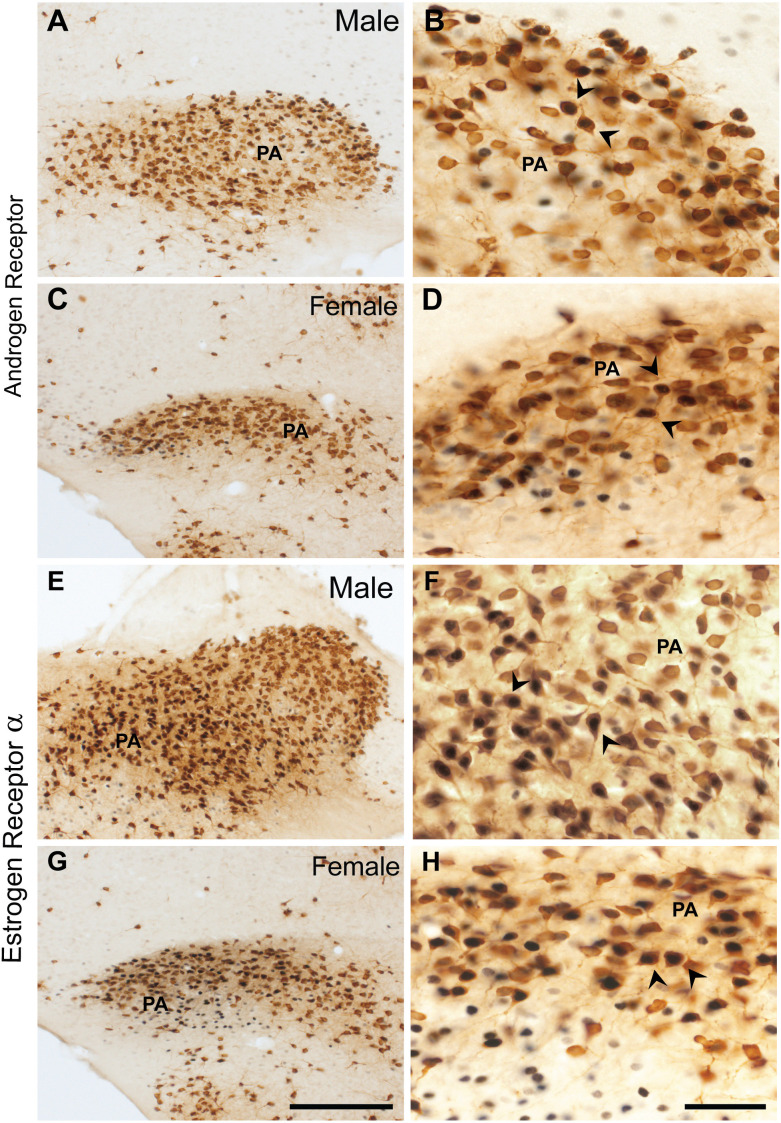
Coexpression of ProkR2-Cre eGFP with AR and in ProkR2-Cre eGFP with estrogen receptor α (ERα) in the posterior nucleus of the amygdala (PA). Bright field micrographs showing the expression of AR (black) and GFP (brown) immunoreactivity in the male PA **(A,B)** and in the female PA **(C,D)**. Bright field micrographs showing the expression of ERα (black) and GFP (brown) in the male PA **(E,F)** and in the female PA **(G,H)**. Arrows in **(B,D,F**, and **H)** indicate dual labeled neurons. Scale Bars: **(A,C,E,G)** = 200 μm; **(B,D,F,H)** = 50 μm.

In males, about 24.7 ± 6.8% of ProkR2-Cre eGFP cells expressed AR (Figures 4A,B, Table 2), whereas 70.6 ± 7.5% of AR cells were ProkR2-Cre eGFP positive. In contrast, female brains had much lower colocalization (Figures 4C,D, Table 2). About 4.5 ± 1.8% of ProkR2-Cre eGFP cells expressed AR and 22.3 ± 4.3% of AR cells coexpressed ProkR2-Cre GFP.

Similar coexpression between ProkR2-Cre eGFP and ERα was observed in males and females PA (Figures 4E–H, Table 2). About 21.8 ±2.6% of ProkR2-Cre eGFP cells in males coexpressed ERα while 23 ± 2.5% of ProkR2-Cre eGFP cells in females coexpressed ERα. Of note, about 70% of ERα in males were ProkR2-Cre eGFP cells, while about 50% of ERα-ir neurons coexpress ProkR2-Cre eGFP in the female PA (Table 2).

**Figure 5:**
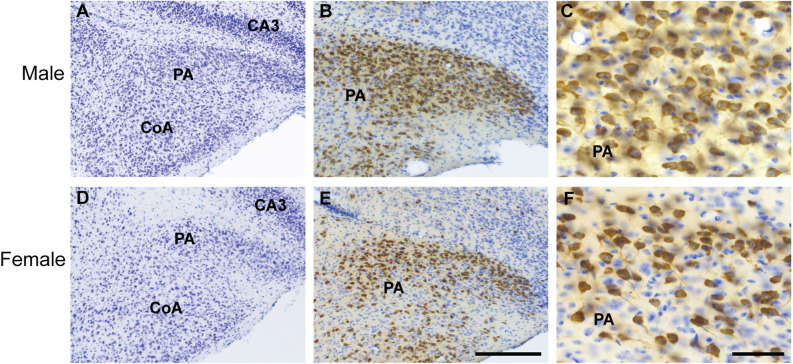
Cytoarchitecture and distribution of ProkR2-Cre eGFP neurons of the posterior amygdala (PA) of male and female brains. Brightfield micrographs showing expression of GFP (brown) and thionin positive cells (blue) in the male **(A–C)** and female PA **(D–F)**. Abbreviations: CoA, Cortical amygdala; CA3, field CA3 hippocampus. Scale Bars: **(A,C)** = 200 μm; **(B,D)** = 50 μm.

To assess if the PA of males and females shows a different number of neurons, we quantified the total number of cells using thionin staining at the level colocalizations were reported (Allen Brain Atlas, level 80, *N* = 3, male and female). No differences were observed. We found that males had on average 463.67 ± 11.7 cells and females 491.33 ± 45.41 in the PA (*P* > 0.05, Figures 5A–F).

## Discussion

In the present study, we performed a systematic evaluation of the colocalization of ProkR2-Cre eGFP with AR or ERα immunoreactivity in the forebrain of male and female mice. Previous work from our and other laboratories have characterized the expression of AR in rodents (Simerly et al., 1990; Wood and Newman, 1995b; Lu et al., 1998; Jahan et al., 2015; Cara et al., 2021). AR is highly expressed in the lateral septum, bed nucleus of the stria terminalis, medial and posterior nucleus of the amygdala, and several nuclei of the hypothalamus, including the medial preoptic, the suprachiasmatic, the paraventricular, the arcuate, and the ventral premammillary nucleus AR expression in the female brain is observed in nearly the same sites as male brains, albeit in much lower density (Brock et al., 2015; Jahan et al., 2015). ERα expression has been previously described to be dense in the lateral septum, bed nucleus of the stria terminals, the medial preoptic nucleus, the arcuate nucleus, the medial amygdala, and the posterior nucleus of the amygdala among several other hypothalamic nuclei of both sexes (Simerly et al., 1990; Wood and Newman, 1995b; Österlund et al., 1998; Merchenthaler et al., 2004; Cao and Patisaul, 2013; Brock et al., 2015). Interestingly, most of the sites expressing these sex steroid receptors are also sites that express *Prokr2* (Cheng et al., 2006; Mohsen et al., 2017).

Having verified that our immunohistochemistry is consistent with previous reports, we found that out of all forebrain sites expressing ProkR2-Cre, the highest colocalization with AR and ERα was observed in the PA of both sexes. We found, however, a clear sex difference with males showing high rates of ProkR2-Cre eGFP neurons coexpressing AR or ERα. These findings place the PA as the main site by which the *Prokr2* neuronal circuit sense and may respond to changes in circulating levels of gonadal steroids potentially modulating reproductive physiology and/or sex and social behaviors. To date, studies investigating the relevance of PROKR2 in reproductive physiology and behaviors have been scarce. The *Prokr2* homozygous null mice exhibited deficits in GnRH neuronal migration and reproductive organ development. The heterozygous null mice, however, were not different from wildtype littermates (Matsumoto et al., 2006). The fertility of the *Prokr2* null heterozygous mice was not assessed, given the normal migration of GnRH neurons, and no behavioral assays were performed in neither female nor male mice. Those heterozygous null mice however exhibited irregular estrous cycles, but the *Prokr2* neural circuitry associated with this observation remains unknown.

The PA is an understudied site as limited publications have focused on the circuitry, chemical phenotype, and functionality of its neurons. Most of the literature on the amygdala’s role in sex and social behaviors has focused on the subdivisions of the medial amygdala. Extensive literature looking at the effects of lesions of the medial amygdala, the projections to and from the medial amygdala, have shown that it plays a critical role in sexual behaviors and aggression, mating and associated behaviors, and it is critical in processing olfactory cues (Kamel et al., 1977; Beltramino and Taleisnik, 1978; Canteras et al., 1995; Wood and Coolen, 1997; Maras and Petrulis, 2010; DiBenedictis et al., 2012; Pardo-Bellver et al., 2012; Bergan et al., 2014; Hari Dass and Vyas, 2014; Keshavarzi et al., 2014; Li et al., 2015, 2017; McCarthy et al., 2017). Few studies have also suggested that the PA is associated with chemosensory pathways related to reproductive behaviors. For instance, studies in Syrian hamsters described that the PA expresses Fos-immunoreactivity, a marker of neuronal activation, in both mating and agonistic behaviors (Kollack and Newman, 1992; Kollack-Walker and Newman, 1995).

Using retrograde and anterograde tracers in rats, studies revealed that innervation of the PA comes from areas associated with processing odor inputs from the vomeronasal system, such as the posterodorsal subdivision of medial amygdala (Canteras et al., 1992). The PA also innervates areas such as the lateral septum, and the bed nucleus of the stria terminalis (Canteras et al., 1992). These findings suggested that the PA is relevant for social behaviors and, together with the medial amygdala, comprises the “olfactory amygdala” (Canteras et al., 1992, 1995; Swanson, 2003).

The murine PA expresses a dense collection of gonadal steroids receptor (Simerly et al., 1990; Wood and Newman, 1995b; Österlund et al., 1998; Merchenthaler et al., 2004; Brock et al., 2015; Jahan et al., 2015; Cara et al., 2021). Specifically, the PA neurons express AR and ERα, and about half of steroid receptor containing neurons expresses both (Wood and Newman, 1995b). In rats, the sites innervated by the PA are also dense in gonadal steroids’ receptors further implicating this nucleus in neural circuitry sensing circulating gonadal steroids (Pfaff and Keiner, 1973; Simerly et al., 1990).

We showed previously that a higher number of *Prokr2* and ProkR2-Cre eGFP expressing cells is observed in the PA of males (Mohsen et al., 2017). This finding invited speculation on the potential role of PA PROKR2 neurons in both sexes. That ProkR2-Cre eGFP cells of the PA of male and female mice coexpress gonadal steroid receptors further emphasize a reproductive relevant role for these neurons. Sex differences and the role of gonadal steroids’ receptors in a specific subpopulation of PA PROKR2 expressing cells need further investigation.

Expression of the ligand, prokineticin 2, has been characterized in mice and in the macaque monkey brain (Cheng et al., 2006; Burton et al., 2016). Expression patterns were similar in both species. The areas known to highly express prokineticin 2 relevant to reproduction are the olfactory bulb, the medial amygdala, the medial preoptic area, the arcuate nucleus, and the suprachiasmatic nucleus (Cheng et al., 2006). Whether prokineticin 2 neurons in these or alternative brain sites project to PA is not known. Additional studies are needed to inform which prokineticin 2 expressing sites innervate the PA.

Use of genetic manipulations of PA neurons including remote control (i.e., opto or chemogenetic) is very limited but in recent years, a study in male mice showed that a subpopulation of these neurons coexpresses vesicular glutamate transporter 1 (Vglut1). The PA Vglut1 neurons project to the ventrolateral subdivision of the ventromedial nucleus of the hypothalamus and induce aggression when activated (Zha et al., 2020). Another study identified two subpopulations of ERα PA neurons, one that projects to the medial preoptic nucleus and another that projects to the ventromedial hypothalamus (Yamaguchi et al., 2020). The first was activated during mating and was necessary and sufficient for sexual behaviors in males and the second was activated during intermale aggression (Yamaguchi et al., 2020). Collectively, these studies indicate that aggression and mating behavior are controlled by a subset of ERα PA cells. In both studies, it is likely that PROKR2 expressing cells were also activated, given that *Prokr2* gene is enriched in the PA (Yamaguchi et al., 2020) and that a subpopulation of PROKR2 PA neurons express ERα. Projection profiles and remote control of PROKR2 expressing cells need further investigation to define the contribution of PROKR2 expressing cells to these behaviors. In addition, the role of AR in PA PROKR2 expressing cells of males and females is unknown.

The relevance of PROKR2 system in the development of the reproductive axis has been described in both humans and mouse models (Ng et al., 2005; Matsumoto et al., 2006; Hardelin and Dodé, 2008). While this study does not assess the function of PROKR2 within the PA, it begins to unravel the components required for a role in sexual and/or social behavior, i.e., the potential to sense changes in circulating gonadal steroids in conspecific opposite sex mates. Further studies aiming at defining the associated neural circuitry and specific action of PROKR2/AR and/or PROKR2/ERα in reproductive function in both sexes are warranted.

## Data Availability Statement

The original contributions presented in the study are included in the article, further inquiries can be directed to the corresponding author.

## Ethics Statement

The animal study was reviewed and approved by IACUC, University of Michigan (protocol # PRO00010420).

## Author Contributions

BC-L and CE designed the study and contributed to manuscript revisions. BC-L carried out the experiments, analyzed data and compiled the manuscript. All authors contributed to the article and approved the submitted version.
